# Aryl-alcohol oxidases: catalysis, diversity, structure–function and emerging biotechnological applications

**DOI:** 10.1007/s00253-025-13538-7

**Published:** 2025-06-25

**Authors:** Paula Cinca‑Fernando, Aurora Vázquez-Rodríguez, Juan Mangas‑Sánchez, Patricia Ferreira

**Affiliations:** 1 Departamento de Bioquímica y Biología Molecular y Celular, Facultad de Ciencias, Universidad de Zaragoza, Zaragoza, Spain; 2 Instituto de Biocomputación y Física de Sistemas Complejos, BIFI (GBsC-CSIC Joint Unit), Universidad de Zaragoza, Saragossa, Spain; 3 Department of Organic and Inorganic Chemistry, IUQEM, University of Oviedo, Julián Clavería 8, Oviedo, 33006 Spain

**Keywords:** Oxidases, Biocatalysis, Aldehydes, GMC oxidorreductases, Enzyme diversity, Green chemistry

## Abstract

**Abstract:**

Aryl-alcohol oxidases (AAOs) are flavin-dependent enzymes of the glucose-methanol-choline (GMC) oxidoreductase superfamily that catalyze the oxidation of a broad range of activated primary alcohols into their corresponding aldehydes, generating hydrogen peroxide. While traditionally studied in wood-decaying fungi, AAOs have recently been identified in bacteria and arthropods, revealing unexpected structural and functional diversity. These enzymes display broad substrate promiscuity, with preferences shaped by differences in active-site architecture and physicochemical properties. Structural studies across kingdoms show a conserved GMC fold with specific adaptations in substrate-binding domains. Detailed mechanistic insights—particularly from the AAO from *Pleurotus eryngii*—suggest a consensus hydride transfer mechanism involving conserved histidine residues, enabling both oxidase and dehydrogenase activity. To explore AAO diversity, BLAST-based mining was performed across fungal, bacterial, and arthropod genomes, leading to the identification and classification of hundreds of putative AAO sequences. These have been further grouped into distinct structural and evolutionary types based on conserved motifs and active-site architecture, revealing convergent strategies and potential functional specialization across kingdoms. Beyond their natural role in biomass degradation, AAOs hold significant biotechnological potential in green chemistry, including the synthesis of valuable aldehydes, bioplastics precursors like 2,5-furandicarboxylic acid, and applications in asymmetric synthesis. Recent advances demonstrate the feasibility of integrating AAOs into industrial biocatalytic processes and artificial cascades. This growing understanding of AAO diversity, structure–function relationships, and biotechnological applications paves the way for the development of novel sustainable biocatalysts in chemical, pharmaceutical, and material industries.

**Key points:**

- *Aryl-alcohol oxidases (AAOs) occur across fungi, bacteria, and arthropods, with distinct structural and functional features.*
- *Sequence similarity searches reveal diverse AAO types with distinct structural and evolutionary traits.*
- *AAOs enable green synthesis of high-value-added bio-based chemicals.*

**Supplementary Information:**

The online version contains supplementary material available at 10.1007/s00253-025-13538-7.

## Introduction

Aryl-alcohol oxidases (AAO; EC 1.1.3.7), also known as veratryl-, benzyl-, salicyl- and aromatic-alcohol oxidases, are flavin-dependent enzymes belonging to the glucose-methanol-choline (GMC) oxidoreductase superfamily. The first AAO was identified in 1960 from cultures of the fungus *Polystictus versicolor* (now *Trametes versicolor*) (Farmer et al. 1960). Since then, numerous AAOs have been identified in the genomes of various wood-decaying fungi (Ruiz-Dueñas et al. 2021; Ferreira et al. 2015a; Hori et al. 2014; Martinez et al. 2009; Floudas et al. 2012; Fernandez-Fueyo et al. 2012). Several of these enzymes have been biochemically characterized, primarily from basidiomycetes such as *Lentinus sajor-caju*, *Pleurotus eryngii (Pe*AAO*)*, *P. ostreatus*, *P. pulmonarius*, *P. sapidus, Bjerkandera adusta (Ba*AAO*)*, *Phanerochaete chrysosporium*, *Ustilago maydis (Um*AAO), *Coprinopsis cinerea (CcAAO)* and *Moesziomyces antarcticus (Ma*AAO*) *(Bourbonnais and Paice 1988; Guillén et al. 1992, 1990; Sannia et al. 1991; Gutiérrez et al. 1994; Varela et al. 2000; Galperin et al. 2016; Romero et al. 2009; Asada et al. 1995; Couturier et al. 2016; Tamaru et al. 2018; Lappe et al. 2021). Characterized AAOs have also been reported in ascomycetes such as *Botrytis cinerea*, *Geotrichum candidum*, *Aspergillus terreus* and *Thermothelomyces thermophiles (Mt*AAO*) (*Goetghebeur et al. 1992; Kim et al. 2001; Chakraborty et al. 2014; Kadowaki et al. 2020*).*

In nature, fungal AAOs play a crucial role in lignocellulose degradation by wood-decaying fungi. Lignocellulose, the most abundant renewable biomass on Earth, primarily comprises cellulose, hemicellulose, and lignin (Bugg 2024). While enzymes readily hydrolyze cellulose and hemicellulose, lignin is a recalcitrant aromatic polymer, resistant to degradation (Janusz et al. 2017; Andlar et al. 2018). Lignin decomposition requires the action of several oxidoreductases such as laccases, which depend on molecular oxygen (O_2_) and peroxidases—including lignin peroxidases, manganese peroxidases and versatile peroxidases—that utilize hydrogen peroxide (H_2_O_2_) as the oxidant. In this scenario, AAOs facilitate lignin breakdown by providing the necessary H_2_O_2_, functioning alongside accessory enzymes like glyoxal oxidases, copper-radical oxidases, and other flavoenzymes from GMC superfamily (Guillén et al. 1990; Martínez et al. 2005). Notably, GMC proteins share with other flavoproteins an ADP-binding domain involved in stabilizing the FAD cofactor, as well as two specific consensus motifs —PS00623 and PS00624— located in the N-terminal region and the central portion of these enzymes, respectively (Cavener 1992).

AAOs catalyze the oxidation of a broad range of activated primary alcohols—particularly benzyl and allylic alcohols—into their corresponding aldehydes, concurrently reducing O_2_ to H_2_O_2_. Unlike oxygenases, AAOs use O_2_ as the preferred electron acceptor and do not require nicotinamide cofactors (Urlacher and Koschorreck 2021). This feature, along with their stability, broad substrate specificity, and high selectivity, underscores the potential of AAOs as valuable biocatalysts for industrial aldehyde synthesis. Aldehydes are key intermediates in synthetic chemistry, with special relevance in asymmetric transformations (Erkkilä et al. 2007; Mukherjee et al. 2007) and are also valuable products in the pharmaceutical, food and cosmetic industries (Heath et al. 2022; Zhu et al. 2018). In this context, fungal AAOs have traditionally been the primary focus of application-oriented research due to their generally excellent catalytic properties (Viña-Gonzalez and Alcalde 2020; Carro et al. 2018a; Liu et al. 2020; Ferreira et al. 2005). Additionally, this group of enzymes has shown promise in diverse biotechnological applications, including pulp biobleaching, treatment of industrial effluents, and production of biopolymer precursors (Serrano et al. 2020; Sigoillot et al. 2005; Carro et al. 2015; Ledakowicz and Paździor 2021; Tamboli et al. 2011). However, although fungal AAOs are extracellular enzymes, their production often requires complex eukaryotic expression systems or additional refolding steps when heterologously expressed in *Escherichia coli*, posing challenges for large-scale industrial use.

Although AAOs have been predominantly characterized in fungi, AAO-like enzymatic activities have also been reported in other organisms. In gastropods, such as *Arion ater* (*Aat*AAO), *Helix aspersa*, and *Limax flavus* (Large and Connock 1993; Mann et al. 1989), as well as in arthropods including *Phratora vitellinae*, *Chrysomela populi* (*Cp*AAO), and *C. tremulae* (Brückmann et al. 2002; Michalski et al. 2008*)*, homologous enzymes have been identified. The discovery of bacterial AAOs began with *Sphingobacterium* sp. ATM (*Satm*AAO) in 2011 (Tamboli et al. 2011), which sparked growing interest in bacterial sources and highlighted their functional diversity and ecological significance. Subsequent characterization of AAOs from *Streptomyces hiroshimensis* (S*h*AAO) and *Sphingobacterium daejeonense* (*Sd*AAO) (Cinca-Fernando et al. 2024) has further expanded our understanding of these enzymes in prokaryotes, offering promising alternatives to address the challenges associated with fungal AAO production.

## AAO substrate scope and biocatalytic properties

**Fig. 1:**
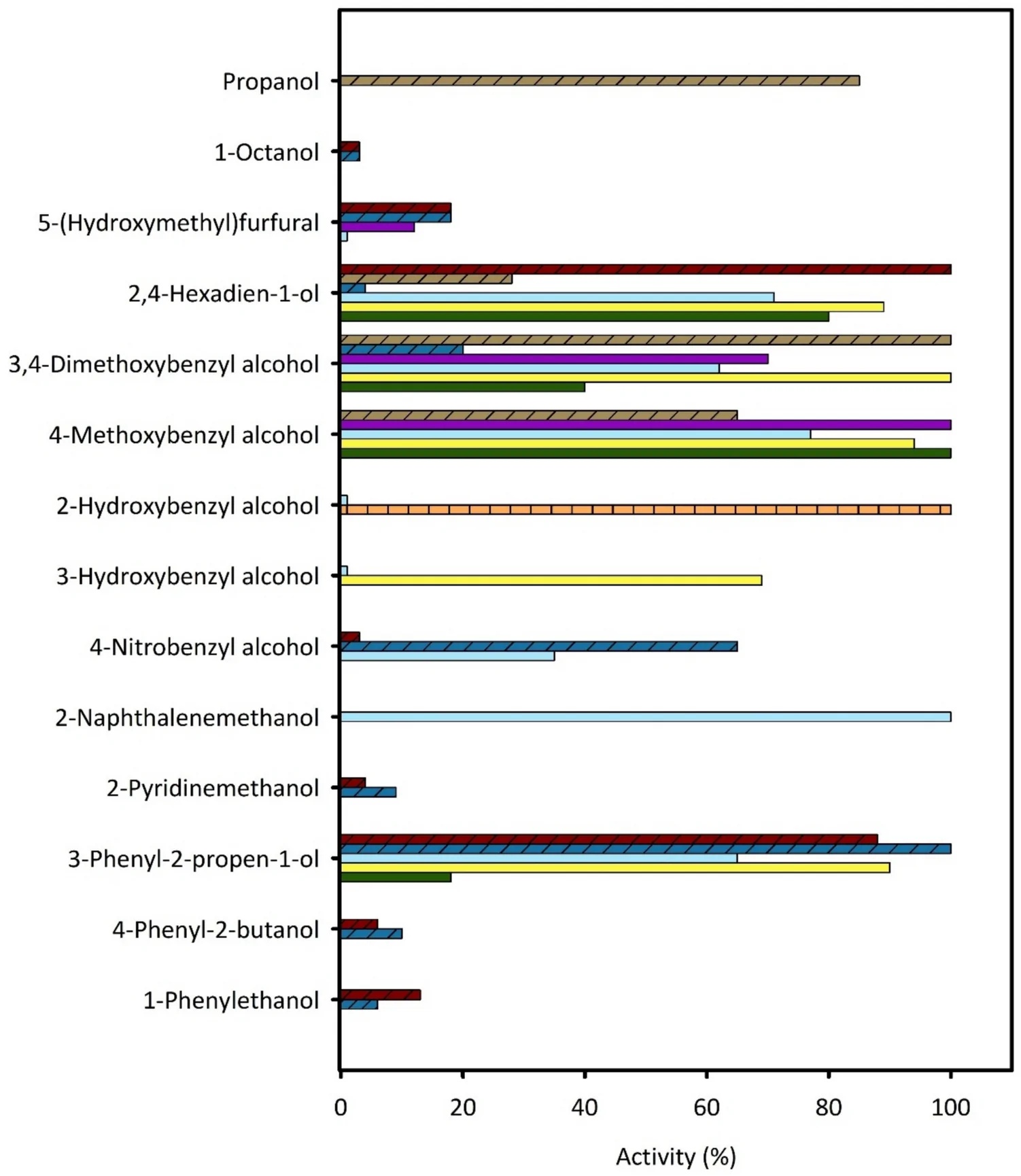
Substrate scope from AAO from *C. populi* (orange), *B. adusta* (green), *C. cinerea* (yellow), *P. eryngii* (light blue), *T. thermophilus* (purple), *S. daejeonense* (dark blue), *Sphingobacterium* sp. (brown), *S. hiroshimensis* (dark red). Fungal AAOs are shown in solid colors, bacterial AAOs are represented with diagonal hatching, and arthropod AAOs with vertical hatching. Relative activities are referred to the best substrate of each enzyme as 100%

AAO enzymes can oxidize a broad range of alcohols, including both phenolic and non-phenolic aryl-alcohols, as well as polyunsaturated aliphatic primary alcohols and aromatic secondary alcohols (albeit with significantly lower efficiency). The substrate promiscuity of fungal AAOs is typically attributed to their extracellular ligninolytic nature, as they utilize secondary fungal metabolites—such as 4-methoxybenzyl alcohol and other benzylic alcohols—alongside various lignin-derived compounds as natural substrates. However, substrate preferences vary across AAOs from different sources (Fig. 1). The activity on aromatic alcohols is influenced by the position and the electronic properties of substituents on the aromatic ring. Generally, fungal AAOs exhibit higher catalytic activity (in terms of turnover rates or initial velocities) toward electron-donating substituents, such as methoxy groups, which facilitate hydride transfer from the benzylic position to the flavin cofactor. Conversely, electron-withdrawing substituents, such as nitro groups, negatively affect enzymatic activity. β-Naphthyl methanol is one of the preferred aromatic alcohol substrates for *Pleurotus* AAOs, followed by 4-methoxybenzyl alcohol and 3-phenyl-2-propen-1-ol (Guillén et al. 1992; Jankowski et al. 2020). *Ba*AAO shows a preference for mono- and dichlorinated anisyl alcohols, which are physiologically secreted by this basidiomycete (de Jong et al. 1994; Romero et al. 2009; Ferreira et al. 2023; Gutiérrez et al. 1994). Both *Ba*AAO and *Ma*AAO efficiently oxidize vanillyl alcohol at rates comparable to those observed with 3,4-dimethoxybenzyl alcohol (Romero et al. 2009; Lappe et al. 2021). *Cc*AAO exhibits higher activity toward hydroxybenzyl alcohols than other fungal AAOs (Tamaru et al. 2018). Polyunsaturated aliphatic alcohols, such as 2,4-hexadien-1-ol and 2,4-heptadien-1-ol, are also good substrates for many fungal AAOs, whereas their non-conjugated analogues are less efficiently oxidized (Guillén et al. 1992; Tamaru et al. 2018; Jankowski et al. 2020). This trend in substrate specificity was also observed in the recently identified bacterial *Sh*AAO, which prefers 2,4-hexadien-1-ol and 3,7-dimethyl-2,6-octadien-1-ol, followed by 3-phenyl-2-propen-1-ol (Cinca-Fernando et al. 2024). In contrast, typical benzylic alcohols are not accepted as substrates. Bacterial *Sh*AAO and *Satm*AAO also display activity towards saturated aliphatic primary alcohols such as 1-propanol and 1-octanol (Cinca-Fernando et al. 2024; Tamboli et al. 2011). On the other hand, bacterial *Sd*AAO preferentially oxidizes aromatic primary alcohols, showing the highest activity with 3-phenyl-2-propen-1-ol, followed by benzylic alcohols bearing electron-withdrawing groups such as 4-bromobenzyl and 4-nitrobenzyl alcohols. Interestingly, 3-phenyl-2-propen-1-ol is also a preferred substrate of ascomycete *Mt*AAO, suggesting that the propene substituent does not cause steric hindrance in these AAOs, likely due to wider substrate channels that facilitate access to the active site (Cinca-Fernando et al. 2024; Kadowaki et al. 2020) (see next section for further details). These findings suggest that differences in substrate specificity among AAOs are largely determined by the size, accessibility, and physicochemical properties of their active sites, which are influenced by amino acid composition.

In addition to their primary alcohol-oxidizing function, several fungal AAOs—such as *Pe*AAO and *Ba*AAO—and *Aat*AAO from *A. ater* have been shown to oxidize aldehydes to their corresponding acids (Romero et al. 2009; Ferreira et al. 2010; Guillén et al. 1992; Mann et al. 1989; Large and Connock 1993). Although this activity is less efficient than their alcohol oxidation, it likely proceeds via oxidation of gem-diols formed upon spontaneous aldehyde hydration (Ferreira et al. 2010). Therefore, AAO enzymes are capable of producing of 2,5-furandicarboxylic acid (FDCA) —a renewable alternative to petroleum-derived terephthalate used in polymer manufacturing— from 5-hydroxymethylfurfural (HMF) through a sequence of three consecutive oxidation steps (Serrano et al. 2019a; Carro et al. 2018b).

Fungal AAOs have been traditionally regarded as highly efficient oxidases, with catalytic constants (*k*_cat_/*K*_m_) for their best substrates reaching ~ 10^6^ M^−1^ s^−1^, comparable to those for glucose oxidases, another GMC protein, considered as “the Ferrari of the oxidases” (Mattevi 2006). Interestingly, several fungal AAOs —such as *Pe*AAO, *Ba*AAO (Ferreira et al. 2023), and *Um*AAO (Couturier et al. 2016)— have also demonstrated quinone reductase activity, similar to other GMC superfamily members. Notably, *Pe*AAO and *Ba*AAO display catalytic efficiencies as dehydrogenase that rival or even exceed their oxidase activities, suggesting that both O_2_ and quinones may act as natural electron acceptors. These classical AAOs differ from described aryl-alcohol quinone oxidoreductases from *Pycnoporus cinnabarinus*, which display predominant quinone reductase activity with negligible oxidase function (Mathieu et al. 2016). These findings support a physiological role for AAO-mediated quinone reduction in lignin degradation and open new avenues for their application in bioelectrocatalysis.

## Structural insights into the aryl-alcohol oxidase family

Structural characterization has been essential in understanding the diversity of the AAO family, providing deeper insights into structural variations and functional mechanisms. For decades, structural data were limited to the crystal structure of *Pe*AAO from *Pleurotus eryngii*, initially solved in its free form and later in complex with *p*-anisic acid —the final product after complete oxidation of its natural substrate— (Fernández et al. 2009; Carro et al. 2017). In 2020, the crystal structure of *Mt*AAO from *T. thermophilus*, a thermophilic biomass-degrading ascomycete, was resolved (Kadowaki et al. 2020). More recently, additional structures were elucidated, including *Ba*AAO from another basidiomycete, *B. adusta*, also in complex with *p*-anisic acid, as well as the first bacterial AAOs, i.e., *Sh*AAO from *S. hiroshimensis* and *Sd*AAO from *S. daejeonense*, both co-crystallized with substrate analogues (Serrano et al. 2024; Cinca-Fernando et al. 2024).

**Fig. 2:**
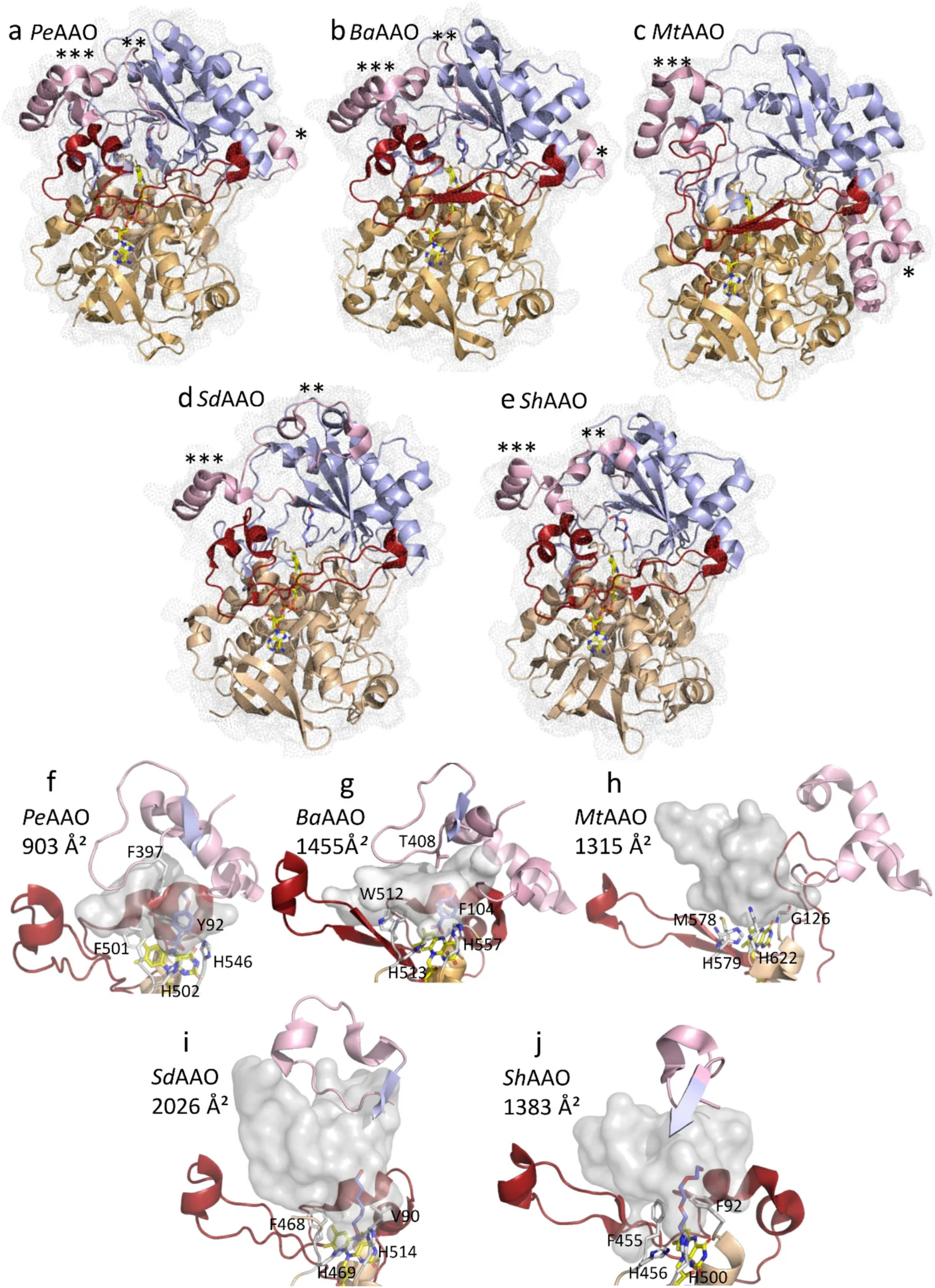
Comparative analysis of overall crystal structures and active-site accessibility. Panels **a**, **b**, **c**, **d**, and **e** represent the crystal structures of *Pe*AAO (PDB ID: 5OC1), *Ba*AAO (PDB ID: 9AVH), *Mt*AAO (PDB ID: 6O9C), *Sd*AAO (PDB ID: 8RPF), and *Sh*AAO (PDB ID: 82PG), respectively. Structures are shown in cartoon representation overlaid with transparent dotted molecular surfaces. The substrate-binding domains are highlighted in light blue, the FAD-binding domains in wheat, and the elongated unstructured elements connecting both domains in red. Structural elements differentiating AAO within the GMC superfamily are depicted in pink. The symbol * indicates a structural element interacting laterally with the FAD-binding domain. Symbols ** and *** indicate additional connections between the FAD-binding and substrate-binding domains unique to the AAO family, which likely modulate active-site accessibility. Panels **f**, **g**, **h**, **i**, and **j** represent the substrate binding sites and access channels to the active sites, including highly conserved histidine residues and those limiting active site accessibility, of the crystal structures of *Pe*AAO (PDB ID: 5OC1), *Ba*AAO (PDB ID: 9AVH), *Mt*AAO (PDB ID: 6O9C), *Sd*AAO (PDB ID: 8RPF), and *Sh*AAO (PDB ID: 82PG), respectively. The access channels and active-site pockets are shown as grey surfaces (calculated by HOLLOW; Ho and Gruswitz (2008)). The FAD cofactors are represented as sticks with carbons in yellow, while alcohol molecules (bacterial AAOs) and the 4-methoxybenzoic (*Pe*AAO and *Ba*AAO) are displayed with blue carbons

Comparative analyses reveal a conserved folding topology among all GMC superfamily members, consisting of a highly conserved N-terminal domain involved in FAD-binding, and a less conserved C-terminal domain with variable sequence and structural elements responsible for substrate recognition and binding (Cavener 1992) (Fig. 2a–e). Among fungal AAOs, *Pe*AAO and *Ba*AAO share a high degree of structural similarity, with an RMSD of 1.2 Å for 578 Cα. *Mt*AAO shows moderate similarity, with RMSD values of 2.5 Å and 2.6 Å compared to *Pe*AAO and *Ba*AAO, respectively (over 613 Cα atoms). Among bacterial AAOs, *Sh*AAO and *Sd*AAO display an RMSD of 2.0 Å across 518 Cα atoms, with *Sh*AAO showing slightly greater similarity to fungal AAOs (RMSD of 1.6 Å with *Pe*AAO and 1.7 Å with *Ba*AAO).

Additionally, the linker segments connecting the N- and C-terminal domains are a major source of structural variation within the AAO family (highlighted in red in Fig. 2). In *Pe*AAO and the bacterial enzymes, these regions consist of long, unstructured loops, whereas in *Ba*AAO and *Mt*AAO, they adopt a defined two-stranded antiparallel β-sheet conformation.

The FAD-domain adopts a Rossmann fold featuring the canonical βαβ motif that accommodates the ADP moiety of the FAD cofactor. It includes the conserved GMC motif G-X-G-X-X-G-(X)18-E as a key structural element. In AAOs, FAD is non-covalently bound in an extended conformation, stabilized by hydrogen bonds with the βαβ motif and surrounding water molecules. While the adenine moiety of FAD is deeply buried, the isoalloxazine ring—particularly the N5 atom involved in redox catalysis—shows variable exposure depending on the accessibility of the active site through hydrophobic channels.

The substrate-binding domain consists of a four- to six-stranded antiparallel β-sheet flanked by a variable number of α-helices, with two highly conserved histidine residues located on the *re* side of the isoalloxazine ring, identified as catalytic residues in *Pe*AAO (H502 and H546) (Fig. 2f) (Hernández-Ortega et al. 2012c; Carro et al. 2018c). Despite overall structural similarity, notable differences exist in three key components within the substrate-binding region (highlighted in light pink, Fig. 2f–j). Fungal AAOs feature an additional segment forming a short α-helix in *Pe*AAO and *BaAAO*, while in *Mt*AAO, this region includes two extended α-helices, resulting in an expanded structures compared to bacterial counterparts (* in Fig. 2a–e). Two additional insertions (** and *** in Fig. 2a–e) form a structural platform above the active site that modulates substrate access (Figure S1). In *Ba*AAO, and particularly *Pe*AAO, a cluster of three α-helices and a 14-residue loop —including Thr498 (*Ba*AAO) and F397 (*Pe*AAO)— creates a narrow, hydrophobic funnel that restricts solvent access to the active site (Fig. 2f–g) (Fernández et al. 2009; Hernández-Ortega et al. 2011a; Serrano et al. 2024). Furthermore, aromatic residues, Y92 and F501 in *Pe*AAO (F104 and W512 in *Ba*AAO), create a bottleneck that limits substrate access and mediates hydrophobic interactions with the ligand (Fig. 2f–g).

In contrast, bacterial AAOs present this structural motif in a more open arrangement. The corresponding loops partially fold into short α-helices but do not significantly obstruct access to the active site, particularly in *Sd*AAO, which displays the most open conformation among the characterized AAOs (Fig. 2i) (Cinca-Fernando et al. 2024). Nevertheless, both *Sh*AAO and *Sd*AAO maintain a conserved active site cavity located on the *re* side of the FAD, lined by hydrophobic residues (V90, F468, H469, H514 in *Sd*AAO; F92, F455, H456, H500 in *Sh*AAO), creating a nonpolar environment conducive to substrate stabilization.

The active site of *Mt*AAO, similar to other GMC enzymes, is fully solvent-accessible due to the absence of the unstructured insertion and a more open conformation of the surrounding elements. Additionally, the *Mt*AAO structure uniquely features a Ca^2^⁺ binding site near the entrance of the catalytic cavity —a structural element not observed in previously reported GMC proteins— which likely contributes to its enhanced thermostability (Kadowaki et al. 2020).

## Catalytic mechanism of aryl-alcohol oxidases

Understanding the molecular basis of a biocatalyst’s activity is essential for expanding its biotechnological applications. In this context, the catalytic mechanism of *Pe*AAO has been extensively studied, establishing it as a model mechanism not only for the AAO family but also for the entire GMC oxidoreductase superfamily.

Computational studies of ligand migration into *Pe*AAO active site have demonstrated that substrate diffusion requires conformational changes in the aforementioned loop which restricts access to the catalytic pocket (Hernández-Ortega et al. 2011a). The swinging motion of the alcohol substrate, facilitated by residue F397, is of particular relevance. Alongside interactions involving residues Y92 and F501, F397 plays a key role in orienting the substrate into a catalytically competent position, placing its α-carbon near the FAD isoalloxazine ring and the side chains of H502 and H546 (Carro et al. 2018a; Ferreira et al. 2015b).

**Fig. 3:**
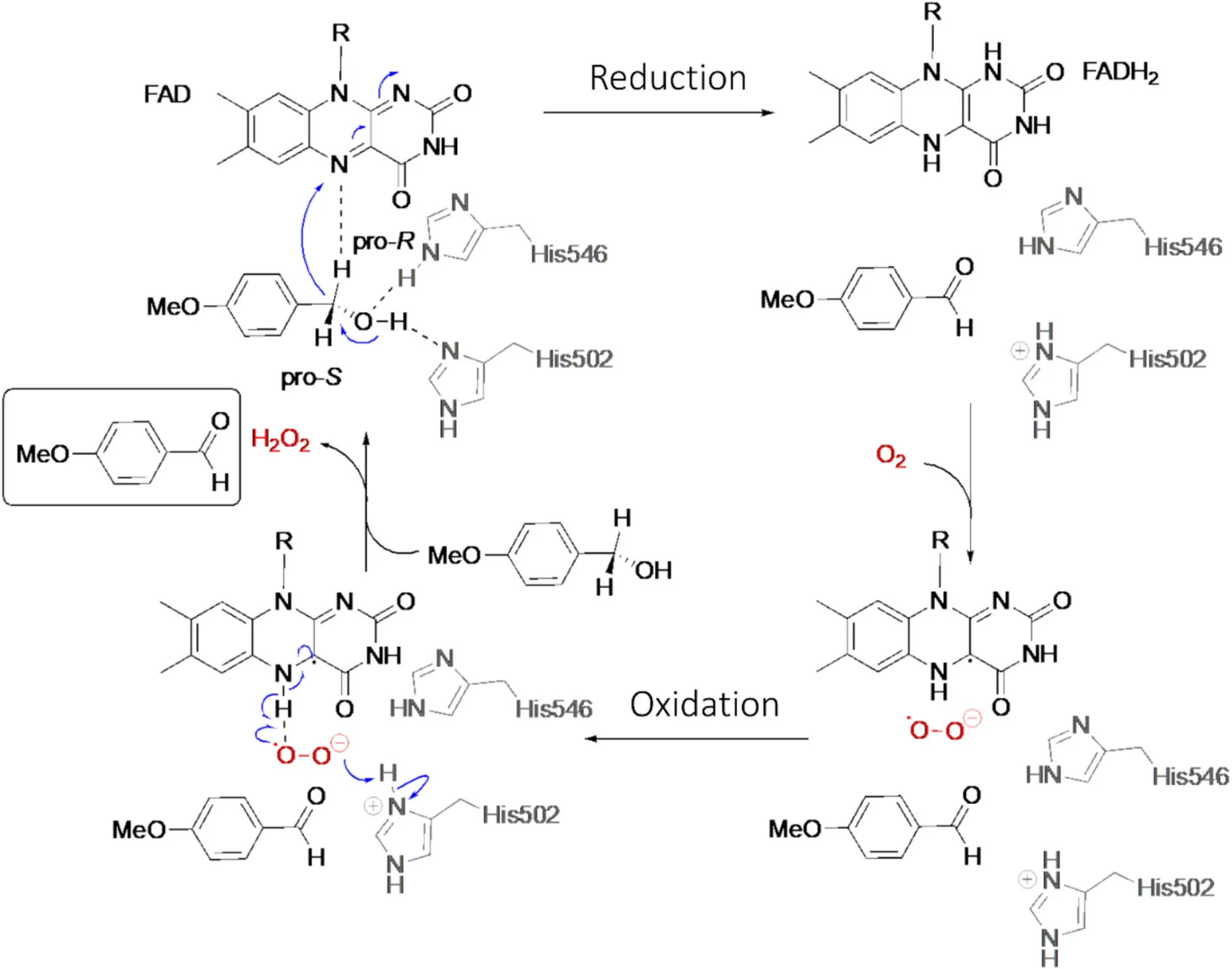
Catalytic cycle of AAO consisting in a reductive half-reaction and an oxidative half-reaction

Upon substrate binding, the AAO catalytic cycle proceeds through two half-reactions—reduction and oxidation—focused on the flavin cofactor (Fig. 3). In the reductive half-reaction, the alcohol substrate undergoes a two-electron oxidation to yield the corresponding aldehyde via a concerted hydride transfer mechanism. This involves simultaneous activation of the alcohol through abstraction of its hydroxyl proton (H⁺) by the catalytic base H502, and selective hydride transfer (H⁻) from the pro-R hydrogen to the flavin N5, forming the reduced flavin in its hydroquinone form (FADH^−^). This oxidative dehydrogenation is facilitated by residue H546, which participates in hydrogen bonding with the substrate (Ferreira et al. 2009; Hernández-Ortega et al. 2012b, 2012c).

In the oxidative half-reaction, the versatile oxidase and dehydrogenase activities of AAO allow for the efficient reoxidation of FADH⁻ via a two-electron transfer to either O₂ or quinones. When O_2_ acts as the electron acceptor, it freely diffuses into the active site and reacts with the reduced flavin to yield a superoxide anion radical and the neutral semiquinone (not thermodynamically stabilized). The final reoxidation step involves the hydride transfer from FAD N5 (originally abstracted from the alcohol substrate in the reductive half-reaction) to the superoxide, along with a proton from the solvent or a solvent-exchangeable site, such as H502, ultimately yielding H_2_O_2_ (Hernández-Ortega et al. 2012c; Carro et al. 2018c). Additionally, residues F501 and F397 modulate the flavin environment by reducing the space in the active site, guiding O_2_ closer to the reactive flavin C4a (Hernández-Ortega et al. 2011b; Carro et al. 2018a). Interestingly, *Mt*AAO, which presents a fully accessible catalytic active-site, exhibits significantly lower O_2_ reactivity, with turnover rates around 200 times lower than those observed for *Pe*AAO (Kadowaki et al. 2020). Recent mechanistic and structural studies on *Ba*AAO align with the overall catalytic mechanism described for *Pe*AAO (Serrano et al. 2024). However, a recent comparative study on their dual oxidase and dehydrogenase activities suggests that quinone diffusion is likely the rate-limiting step, an observation that mirrors findings for glucose oxidase (Serrano et al. 2024; Mtemeri and Hickey 2023). The wider substrate channel of *Ba*AAO may facilitate product release, whereas in *Pe*AAO, displacement of the F397 side chain is required for product exit (Carro et al. 2018a). Furthermore, *Pe*AAO also catalyzes aldehyde oxidation via the same mechanism, acting on the gem-diol form of hydrated aldehydes, a prerequisite for this activity (Ferreira et al. 2010).

Future structure–function studies on bacterial AAO will clarify the implications for catalysis of their unusually wide and accessible active-site tunnels. Current structural data and substrate-like binding mode are compatible with a redox mechanism involving hydride transfer assisted by a catalytic base, consistent with what has been described for other well-characterized members of the GMC oxidoreductase superfamily. Particularly, residue H469 in *Sd*AAO and H456 in *Sh*AAO align with the highly conserved catalytic histidine found in other GMC oxidoreductases, which is responsible for proton abstraction from the alcohol substrate during the reductive half-reaction.

## Exploring the diversity and evolutionary landscape of AAO enzymes

**Fig. 4:**
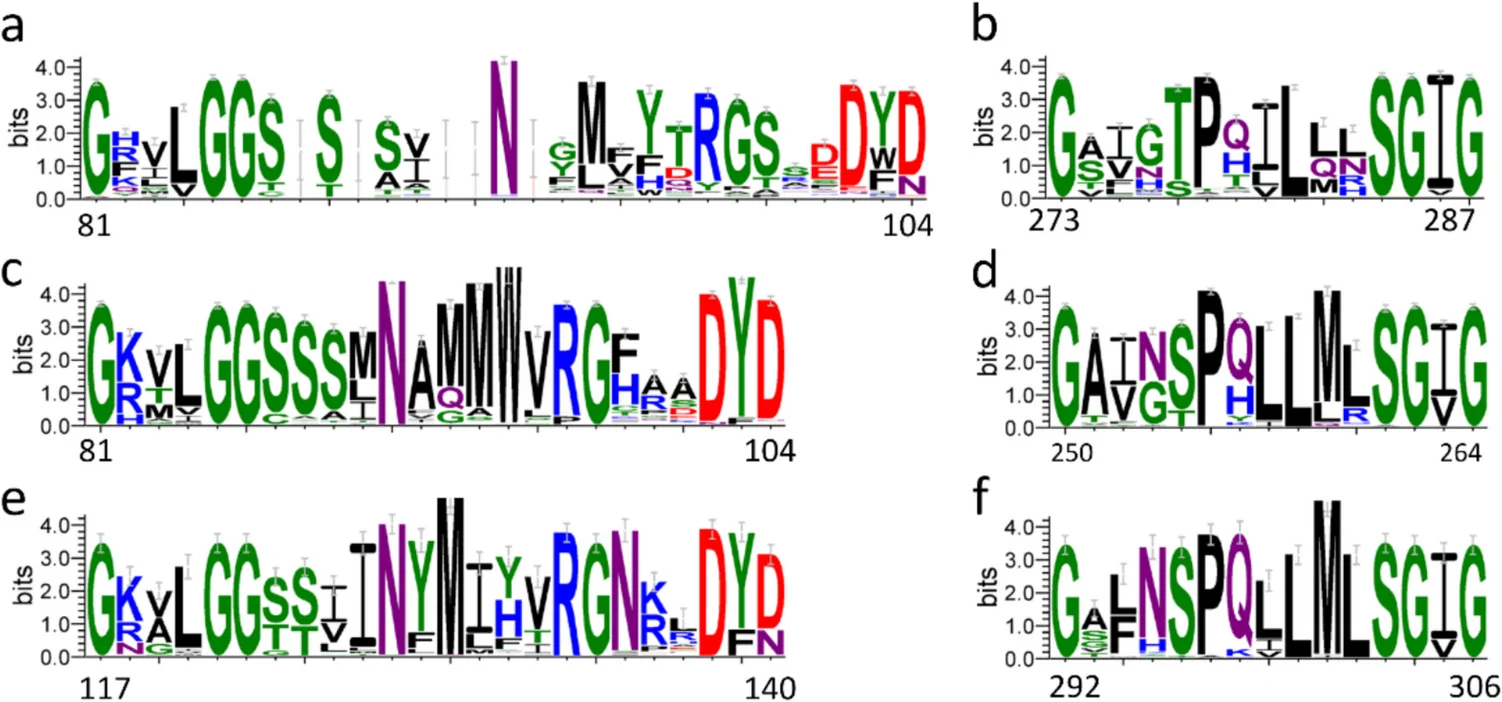
Sequence logo of the conserved motifs typical of GMC proteins corresponding to PS00623 signature ([GA]-[RKNC]-x-[LIVW]-G(2)-[GST](2)-x-[LIVM]-[NH]-x(3)-[FYWA]-x(2)-[PAG]-x(5)-[DNESHQA]) for fungal **a**, bacterial **c** and arthropods AAOs **e**; and PS00624 signature ([GS]-[PSTA]-x(2)-[ST]-[PS]-x-[LIVM](2)-x(2)-S-G-[LIVM]-G) for fungal **b**, bacterial **d** and arthropods AAOs **f**. The numbering indicates the position in *Pe*AAO sequence for fungal logos, in *Sh*AAO for bacterial logos and in *Cp*AAO for arthropods logos. Representation generated using WebLogo3 (Schneider and Stephens 1990; Crooks et al. 2004)

Motivated by the recent discovery and characterization of several bacterial AAOs, along with the distinct features reported for the fungal *Mt*AAO compared to classical Basidiomycetes and other Ascomycetes AAOs, we set out to investigate their distribution across different kingdoms for the identification of potential novel biocatalysts. To achieve this, BLAST searches were conducted against the JGI and NCBI databases using *Sd*AAO and *Sh*AAO sequences to identify bacterial homologues, and *Mt*AAO as a query for fungal homologues. Protein sequences with the highest identity percentages (> 35%) and lowest e-values (< 10^–10^) were selected for further analysis. These candidates were subjected to multiple sequence alignments to identify conserved motifs typical of GMC proteins, focusing on: i) the ADP-binding domain; ii) PS00623 and PS00624 signatures; and iii) two highly conserved catalytic histidine residues characteristic of the AAO family (Fig. 4). Only sequences displaying all these conserved features were retained as putative AAO candidates. Notably, these selection criteria were also applied to the blast searches described above.

In fungi, 93 sequences matching the GMC consensus motifs and catalytic histidines were identified across diverse genera associated with the phylum Ascomycota and were primarily derived from organisms isolated from terrestrial environments such as soil, dung, and decaying wood. To broaden the analysis, we also included previously reported AAO sequences from Basidiomycete genomes (Ferreira et al. 2015a; Sierra-Patev et al. 2023; Ruiz-Dueñas et al. 2021).

In bacteria, 259 sequences were retrieved from a wide range of genera with sources ranging from soil and rhizosphere environments to marine sediments and pathogenic isolates. An additional search was conducted specifically within the genus *Mycobacterium*, given its recent identification as a promising source of enzymes for oxidation reaction (Sayed et al. 2022). This search retrieved an additional 203 sequences derived primarily from strains isolated from human or animal hosts, although several were also obtained from environmental sources such as soil and water.

Finally, considering the presence of AAOs in arthropods, a BLAST search using the salicyl alcohol oxidase (SAO) sequence from *C. populi* as the query was performed (Brückmann et al. 2002). This search identified 91 sequences belonging primarily to Coleoptera species associated with plantations and tree infestations. In nature, SAO enzymes produce salicylaldehyde as a defensive compound in larvae against generalist predators (Pasteels et al. 1983).

Structurally, AAOs across kingdoms display a conserved active site architecture, with substrates located in a cavity on the *re* side of the FAD, adjacent to two conserved histidine residues and several hydrophobic side chains. In *Pe*AAO, two aromatic residues, Y92 and F501, are critical for stabilizing the alcohol substrate, with Y92 engaging in π-π interactions. Interestingly, tyrosine, phenylalanine, and leucine residues are commonly found at the Y92-equivalent position in basidiomycete AAOs but not across broader GMC oxidoreductase superfamily. This observation suggests that aromatic stacking interactions may represent a conserved catalytic strategy for alcohol substrate oxidation in fungal AAOs (Ferreira et al. 2015b). In contrast, recent bacterial AAO and *Mt*AAO crystal structures reveal variability at this position, implying alternative catalytic mechanisms.

**Fig. 5:**
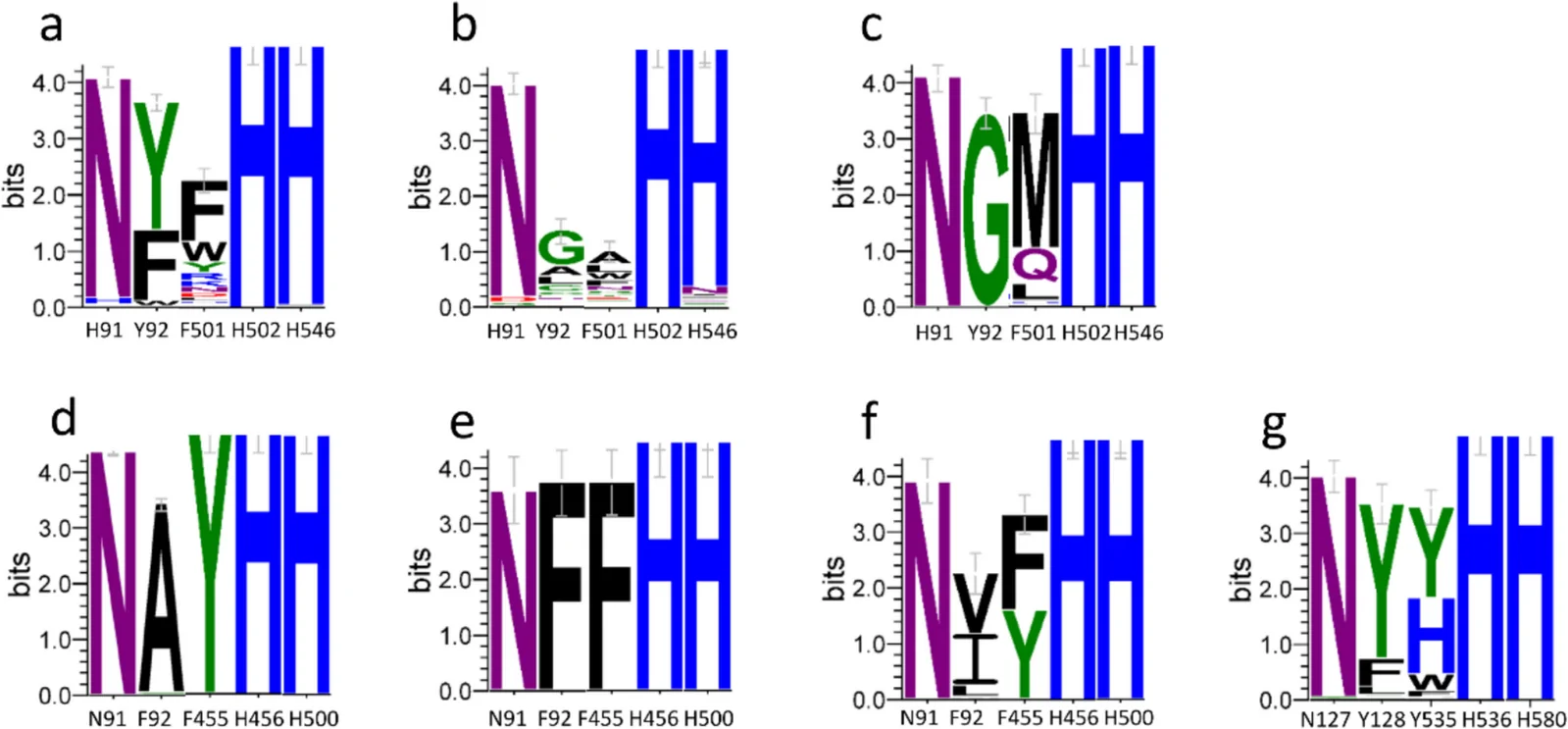
Sequence logo representation of the conservation of the active site residues in fungal type I **a**, type II **b** and type III **c** AAOs, bacterial type I **d**, type II **e** and type III **f** AAOs and arthropods AAOs **g**. The numbering indicates the position in *Pe*AAO sequence for fungal logos, in *Sh*AAO for bacterial logos and in *Cp*AAO for arthropods logos. Representation generated using WebLogo3 (Schneider and Stephens 1990; Crooks et al. 2004)

A comparative analysis of active-site composition allowed us to classify fungal, bacterial, and arthropod AAOs into distinct types, supported by structural modelling using AlphaFold3 (Jumper et al. 2021). Fungal AAOs were categorized into three types based on the aromaticity of residue Y92 in *Pe*AAO. Type I proteins retain an aromatic residue at position Y91 and includes all previously characterized Basidiomycota AAOs (*Pe*AAO, *Ba*AAO, *Cc*AAO, *Um*AAO, *Ma*AAO). In contrast, type II AAOs have lost aromaticity at this position, displaying predominantly aliphatic and uncharged polar residues. Type III, represented by *Mt*AAO and encompassing all AAOs from Ascomycota, is characterized by a highly conserved glycine residue at this position. Regarding position F501 (*Pe*AAO numbering), types I and II retain aromatic residues combined either with polar and charged residues (type I) or with aliphatic and polar residues (type II). In contrast, type III proteins have lost aromaticity at this position, exhibiting only aliphatic and polar residues (Fig. 5).

Structurally, fungal AAOs of types I and II share a common fold, with flexible segments connecting the FAD and substrate-binding domains, sometimes forming a two-stranded β-sheet. Both types include an additional α-helix in the substrate-binding domain. In contrast, type III AAOs possess longer α-helix insertions and a more structured connection, resulting in larger overall dimensions. Active-site accessibility is modulated by a loop structure in types I and II, with type I displaying a consistent 14-residue loop that restricts access (~ 1000 Å^2^ cavity), and type II showing greater variability (12–14 residues; cavities 900–1600 Å^2^). Type III lacks this loop, resulting in a more open cavity (~ 1550 Å^2^).

**Fig. 6:**
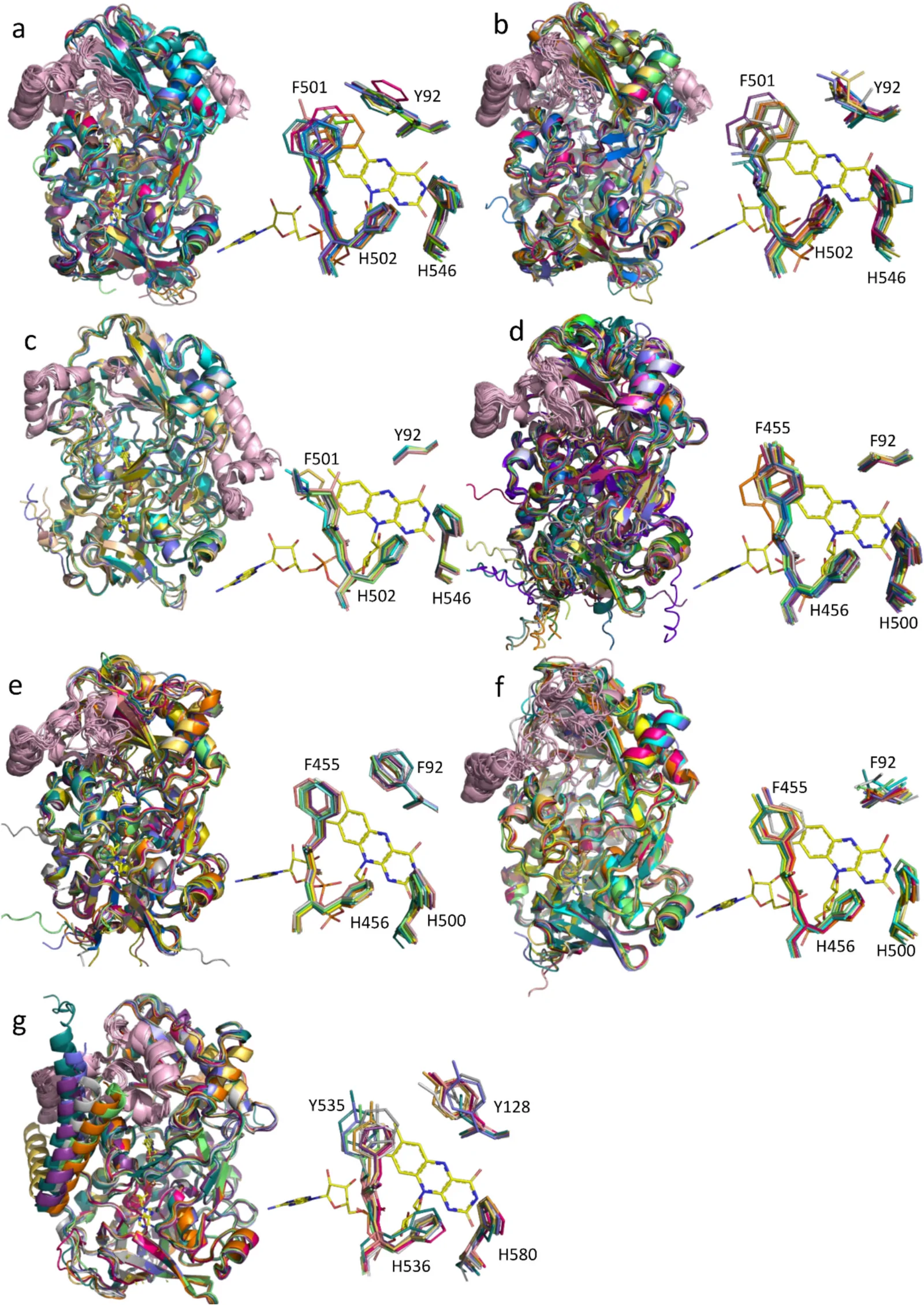
Comparative overview of the AlphaFold3 predicted global structures and active sites for a representative subset of each type of AAO: Fungi type I **a**, Fungi type II **b**, Fungi type I **c**, Bacteria type I **d**, Bacteria type II **e**, Bacteria type III **f** and Arthropods **g**. Global structures are shown in cartoon representation with each structure in a different color. Active sites are shown as sticks and the numbering indicates the residue in *Pe*AAO for fungal structures, in *Sh*AAO for bacterial structures and in *Cp*AAO for arthropods structures. Structural elements differentiating AAO within the GMC family are depicted in light pink. The FAD cofactors are represented as sticks with carbons in yellow

Bacterial AAOs were also grouped into three types based on residue F92 (*Sh*AAO numbering, equivalent to Y92 in *Pe*AAO) and overall structural characteristics. Type I proteins possess an alanine or a glycine residue, type II a phenylalanine, although *Geminicoccaceae* sequences feature a tyrosine instead, and type III an aliphatic residue. Moreover, at position F455 (*Sh*AAO numbering, equivalent to F501 in *Pe*AAO), type I proteins contain a tyrosine, type II a phenylalanine, and type III either a tyrosine or a phenylalanine (Figs. 5 and 6a–c). Based on this classification, *Sh*AAO belongs to type II and *Sd*AAO to type III.

All three bacterial types share a conserved fold and lack the α-helix insertion found in fungal AAOs (Fig. 6d–f). The domain-connecting segment typically consists of long unstructured elements, occasionally forming a two-stranded β-sheet. The most notable differences lie in the loop controlling substrate access to the active site. Type I enzymes exhibit a highly conserved 14-residues loop, similar to that described in *Pe*AAO, resulting in a cavity ~ 1000 Å^2^. In contrast, type II has a shorter 10–12 residue loop (cavity ~ 1500 Å^2^). Type III AAOs exhibit the most variability, with a loop ranging from 8 to 23 residues in length and displaying a variable topology among proteins, leading to significantly larger cavities of up to 2000 Å^2^.

Arthropod AAOs form a single structural type, with conserved aromatic residues at Y128 (*C. populi* numbering, equivalent to Y92 in *Pe*AAO, and predominantly contain tyrosine at Y535 (C. *populi* numbering, equivalent to F501 in *Pe*AAO) or occasionally positively charged residues (Figs. 5 and 6g). These proteins lack the characteristic fungal α-helix insertion in the substrate-binding domain and feature long unstructured elements linking the domains, occasionally forming β-sheets. A distinguishing feature is the replacement of the typical substrate access loop by two helices (~ 32 residues total), yielding a more restricted cavity (~ 850 Å^2^). Arthropod AAOs also have longer overall sequences (~ 600 residues, excluding predicted signal peptides) and a unique N-terminal α-helix.

**Fig. 7:**
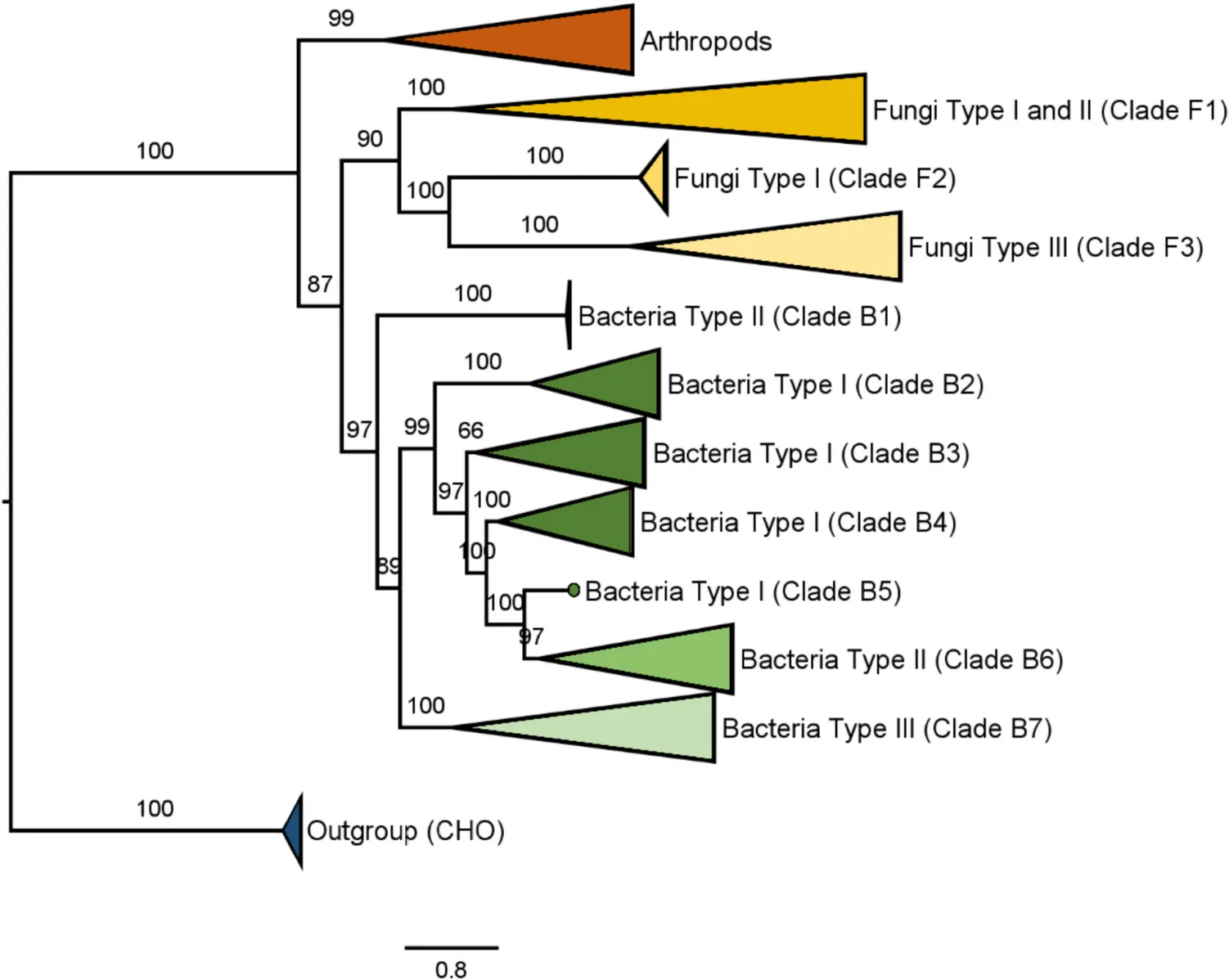
Maximum-likelihood phylogenetic tree incorporating all AAO sequences, as well as three fungal aryl-alcohol quinone oxidoreductases. Multiple sequence alignment was conducted with MUSCLE implemented in MEGA, while the phylogenetic tree was inferred using IQ-TREE, employing the optimal substitution model selected via ModelFinder. Ultrafast bootstrap analysis was performed with 10,000 replicates to assess statistical support. Two cholesterol oxidases from *Gloeocapsopsis* and *Chroococcidiopsis* were used as outgroup. The genera represented within each clade are detailed in Table S1

A comprehensive phylogenetic analysis encompassing all AAO sequences, as well as three fungal aryl-alcohol quinone oxidoreductases (Mathieu et al. 2016), was performed (Fig. 7). The results revealed that arthropod AAOs cluster separately from fungal and bacterial sequences, suggesting a divergent evolutionary origin. Among fungi, types I and II cluster together with fungal dehydrogenases, indicating close evolutionary relationships, while type III AAOs form a separate clade, consistent with divergence between Basidiomycota and Ascomycota lineages. Notably, the characterized AAOs from *M. antarcticus* and *U. maydis*, despite their classification as type I, show close phylogenetic affinity with type III fungal enzymes.

Bacterial sequences formed three well-defined clades corresponding to types I–III. The topology suggests that type II AAOs may have evolved from type I, while type III enzymes likely followed an independent evolutionary path.

## Biotechnological potential

**Fig. 8:**
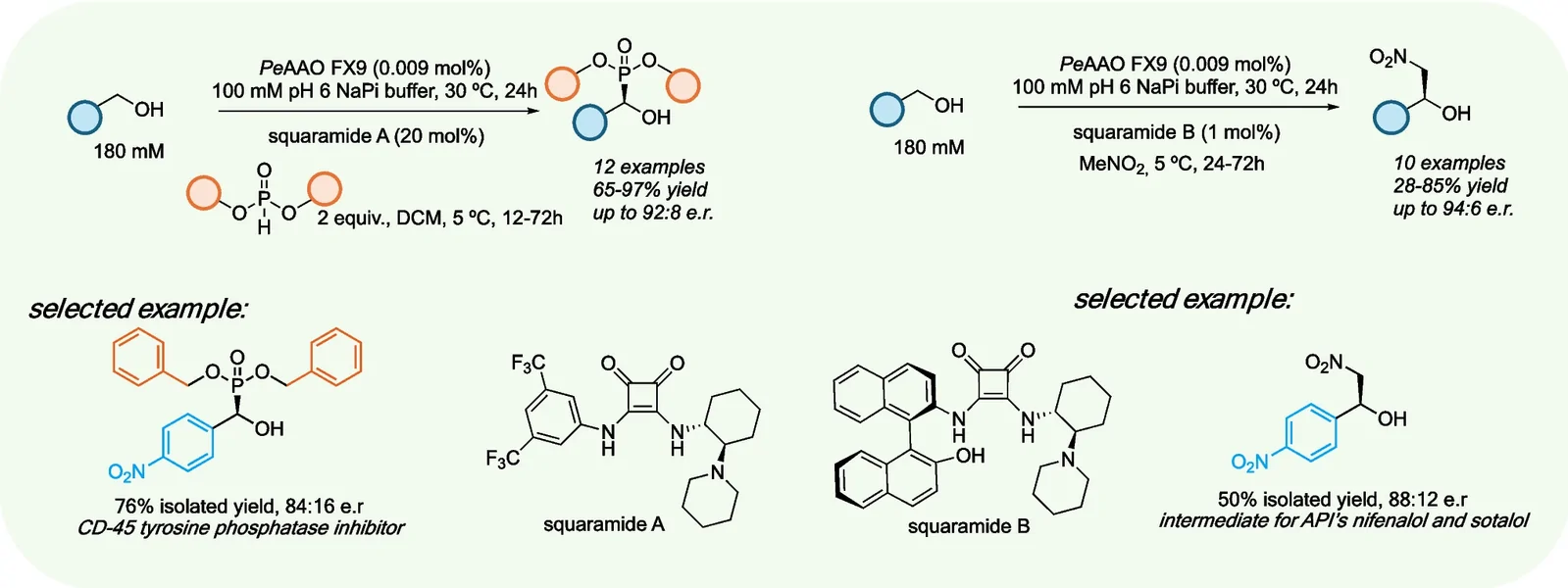
Artificial cascades involving the combination of chemical or enzymatic steps with AAOs for the preparation of chiral synthons

As previously mentioned, a typical AAO reaction involves the oxidation of an alcohol to its corresponding carbonyl compound, coupled with the reduction of molecular oxygen to hydrogen peroxide. This dual functionality has enabled the development of industrial biotechnological applications utilizing both processes. On one hand, AAO-mediated H_2_O_2_ generation has been studied in wastewater treatment to degrade synthetic dyes (Tamboli et al. 2011) and in delignification processes (Sigoillot et al. 2005). On the other hand, the use of AAOs to synthesize industrially relevant compounds represents a higher-value application, offering a sustainable alternative to classical oxidation methods in synthetic chemistry. These methods often involve the use of metals or stoichiometric, hazardous reagents, which can lead to challenges such as overoxidation and complex downstream processing (Dong et al. 2018). To date, different isoforms of *Pe*AAO and its variants have been extensively studied to make biotechnologically relevant compounds. The substrate scope of these enzymes covers the oxidation of a broad range of electron-rich benzylic alcohols as well as aliphatic allylic alcohols such as *trans*−2-hexen-1-ol. The oxidation of this particular alcohol has been the focus of recent research by Hollmann and coworkers, as the resulting aldehyde, *trans*−2-hexenal, is a valuable commodity chemical widely used in the flavor and fragrance industry (de Almeida et al. 2019). One of the main challenges in applying O_2_-dependent reactions to large scale processes in aqueous media is the limited solubility of oxygen in water (ca. 0.25 mM at room temperature). In an initial study, this inherent limitation was addressed by conducting the oxidative process under a continuous flow setup (Van Schie et al. 2018). Using a slug-flow reactor, full conversion was achieved within 40 min starting from 10 mM substrate, corresponding to a turnover number (TN) of 32,000. However, the enzyme exhibited marked substrate inhibition, limiting its efficiency at high substrate concentrations. To address this, the authors explored two-phase systems, where the organic layer serves as a reservoir for both the substrate and product (de Almeida et al. 2019). This approach is effective to lowering substrate concentrations in the aqueous phase, mitigating inhibition issues. Hydrophobic solvents were well tolerated, and dodecane was selected as the organic phase in a 1:1 ratio with the aqueous layer. At 500 mM substrate concentration, the enzyme displayed a remarkable performance, obtaining full conversion with a TN of 650,000. Under neat conditions, a TN of 2,200,000 was obtained after 14 days and several enzyme additions. Jankowski et al., used the AAO 2 from *P. eryngii* P34 (*Pe*AAO2) to synthesize piperonal and other industrially relevant aldehydes after a comprehensive optimization of the reaction conditions at preparative scale (Jankowski et al. 2022). First, the ability of the enzyme to tolerate the presence of organic solvents was investigated, finding relative activities ~ 80% with short-chain alcohols such as methanol or isopropanol, as well as DMSO. A 300 mg-scale reaction (200 mM concentration) using 0.5 µM *Pe*AAO2 was performed in a 100 mL baffled Erlenmeyer flask to improve aeration, obtaining a 95% conversion to piperonal with an 85% isolated yield after crystallization. Under these conditions, other aromatic and allylic primary alcohols were also oxidized in excellent conversions (Fig. 8). Very recently, our group set out to study the potential application of bacterial AAOs in synthetic chemistry (Cinca-Fernando et al. 2024). After initial study of their substrate scope, analytical scale biotransformations were performed using *Sh*AAO as the catalyst at substrate concentrations of up to 80 mM, obtaining high conversions and notable TNs (> 38,000) were obtained with model substrates such as 3-phenyl-2-propen-1-ol and *trans*, *trans*−2,4-hexadien-1-ol, with no overoxidation products detected. The high enzyme expression levels of *Sh*AAO allowed the use of cell-free extracts as biocatalysts, a cost-effective and preferred approach in industry that eliminates the need for additional protein purification steps (Huffman et al. 2019; Cinca-Fernando et al. 2025). Using 40 mM 3-phenyl-2-propen-1-ol as the substrate, cinnamaldehyde was obtained in 86% conversion with no side products detected. Furthermore, a 1 mmol scale reaction was also carried out obtaining cinnamaldehyde in 83% isolated yield after column chromatography, highlighting the synthetic utility of this bacterial AAO.

In recent years, the synthesis of 2,5-furandicarboxylic acid (FDCA) has received increased attention due to its potential for producing polyethylene furanoate (PEF), a biobased alternative to oil-derived plastics such as polyethylene terephthalate (PET). FDCA can be obtained via multistep sequential oxidations starting from 5-hydroxymethylfurfural (HMF), in which the oxidation of the primary alcohol moiety of the different intermediates involved can be catalyzed by AAOs. Different research groups have explored the application of *Pe*AAO and its variants to oxidize HMF to DFF, with subsequent oxidations to the corresponding acid mediated by other enzymes or *Pe*AAO itself (Carro et al. 2018b; Serrano et al. 2019a; Viña-Gonzalez et al. 2020; Karich et al. 2018). Although promising, these reactions have so far been limited to low substrate concentrations (up to 15 mM). Other members of the GMC superfamily showing AAO activity have been specifically identified as HMF oxidases (HMFOs) due to the importance of this compound and the high activities they display towards its oxidation. Early studies focused on enzyme characterization and were restricted to low substrate concentrations (Dijkman and Fraaije 2014; Viñambres et al. 2020). However, a recent study by Tjallinks et al*.* showed that a new HMFO from the honeybee *Apis mellifera* (beeHMFO) was able to convert efficiently HMF concentrations up to 50 mM, demonstrating promising potential for biotechnological applications (Tjallinks et al. 2023).

Another very interesting potential application of AAOs lies on their use in asymmetric chemistry. In *Pe*AAO, the active site architecture enables the stereoselective hydride abstraction from the pro-R proton of the primary alcohol substrate (Hernández-Ortega et al. 2012a). Accordingly, in secondary alcohols, this mechanism theoretically favors hydride abstraction from the *S* enantiomer. Inspired by this concept, Alcalde, Martínez and coworkers developed engineering campaigns to generate *Pe*AAO variants capable of accepting secondary alcohols enantioselectively, thus enabling the kinetic resolution of racemic secondary alcohols (Serrano et al. 2019b; Viña‐Gonzalez et al. 2019). Even though successful variants were obtained, their applications in preparative scale processes has yet to be described.

### Applications in cascade processes

Cascade processes consist in the combination of several consecutive reactions without the purification of the reaction intermediates. In this manner, not only issues associated with product purification are avoided, e.g., organic solvent usage, energy and time consumption, but unstable or toxic reaction intermediates can be quickly converted to other products resulting in safer and more effective synthetic procedures. These processes are particularly convenient and attractive combining multiple biocatalytic processes due to the similar reaction conditions most enzymes present and, since enzymes generally present exquisite selectivity, the risk of cross reactivity and formation of side products is minimized (Rudroff et al. 2018; Schrittwieser et al. 2018; González‐Granda et al. 2023; Ascaso‐Alegre and Mangas‐Sanchez 2022). In this context, alcohol oxidases have been used in different cascade processes to make relevant compounds in combination with other enzymes or chemical steps. Most examples to date involve the use of the *Ac*CO_6_ variant of the FAD-dependent choline oxidase from *Arthrobacter chlorophenolicus* (Heath et al. 2019). These examples clearly demonstrate the potential of alcohol oxidases in synthetic chemistry, enabling the production of a wide range of valuable compounds starting from primary alcohols including amines (Ramsden et al. 2019, 2024), alkenes (Wahart et al. 2024) and chiral γ-nitroaldehydes (Möhler et al. 2024). Concerning the use of AAOs, our group has recently demonstrated that AAOs can also be effectively combined with organocatalysts operating via H-bond catalysis to construct chiral C–C and C-P bonds from primary alcohols (Fig. 8) (Ascaso‐Alegre et al. 2024). In this case, we used a variant of AAO from *P. eryngii*, termed FX9, which proved to be an excellent catalyst for the oxidation of benzylic and aryl allyl alcohols, with TNs > 10,000. For the construction of the asymmetric C-P and C–C bonds, different squaramide-based catalysts were tested in two-phase systems and mild temperatures with yields and stereoselectivities sometimes superior to those displayed in pure organic solvents. After a thorough optimization of both processes, a broad set of 1,2-hydroxyphosphonates and 1,2-nitro alcohols were synthetized starting from benzyl and cinnamyl alcohols in moderate to excellent yields and good enantiomeric ratios. These examples included intermediates in the synthesis of different active pharmaceutical ingredients (APIs). Remarkably, the synthesis of chiral 1,2-nitro alcohols was successfully carried out without the use of additional organic solvents, with MeNO_2_ acting as both the nucleophile and reaction medium. To the best of our knowledge, this remains as the only cascade process combining an AAO with a chemical catalyst to make chiral synthons.

## Supplementary Information

Below is the link to the electronic supplementary material.

ESM 1 (DOCX 1.78 MB)

## Data Availability

No datasets were generated or analysed during the current study.
