# Supplementary material for: Aryl-alcohol oxidases: catalysis, diversity, structure–function and emerging biotechnological applications
Source: Appl Microbiol Biotechnol. 2025 Jun 25;109(1):151. doi: 10.1007/s00253-025-13538-7 (PMC12198266; doi:10.1007/s00253-025-13538-7)
Supplement: Supplementary file 1 — (DOCX 1.78 MB) [file 253_2025_13538_MOESM1_ESM.docx]

**Supporting Information**

**Aryl-alcohol oxidases: catalysis, diversity, structure-function and emerging biotechnological applications**

Paula Cinca‑Fernando^1,2^, Aurora Vázquez-Rodríguez^1^, Juan Mangas‑Sánchez^3^, ·Patricia Ferreira^1,2^

^1^Departamento de Bioquímica y Biología Molecular y Celular, Facultad de Ciencias, Universidad de Zaragoza, Zaragoza, Spain

^2^Instituto de Biocomputación y Física de Sistemas Complejos, BIFI (GBsC-CSIC Joint Unit), Universidad de Zaragoza, Zaragoza, Spain

^3^Department of Organic and Inorganic Chemistry, IUQEM, University of Oviedo, Julián Clavería 8, 33006 Oviedo, Spain

Juan Mangas-Sánchez: mangasjuan@uniovi.es

Patricia Ferreira: ferreira@unizar.es

**Table of Contents**

**Supplementary tables**

**Table S1** Inventory of protein sequences

**Supplementary Figures**

**Figure S1** Comparative analysis of the active-site accessibility

**Supplementary Tables**

| Table S1 Taxonomic composition of the phylogenetic clades identified in the AAO maximum-likelihood tree (Fig. 7). | | | | |
| --- | --- | --- | --- | --- |
| Clade | Genera (nº of sequences) | | | |
| **Arthropods** | *Chrysomela* (12)  *Diabrotica* (10)  *Acanthoscelides* (9)  *Diorhabda* (6)  *Callosobruchus* (4)  *Phaedon* (4)  *Photinus* (4)  *Trypoxylus* (4) | *Abscondita* (3)  *Agrilus* (3)  *Tenebrio* (3)  *Aromia* (2)  *Coccinella* (2)  *Cryptolaemus* (2)  *Ignelater* (2)  *Phratora* (2) | *Psylliodes* (2)  *Tribolium* (2)  *Zophobas* (2)  *Aethina* (1)  *Anthonomus* (1)  *Asbolus* (1)  *Brassicogethes* (1)  *Cylas* (1) | *Dalotia* (1)  *Exocentrus* (1)  *Harmonia* (1)  *Hypothenemus* (1)  *Lamprigera* (1)  *Leptinotarsa* (1)  *Nicrophorus* (1)  *Phyllotreta* (1) |
| **Fungi Type I and II** (Clade F1) | *Pholiota* (42)  *Pleurotus* (41)  *Coprinopsis* (37)  *Gymnopus* (34)  *Lentinula* (31)  *Cyathus* (27)  *Lepista* (22)  *Marasmius* (22)  *Clitocybe* (20)  *Armillaria* (20)  *Volvariella* (18) | *Macrolepiota* (17)  *Hymenopellis* (16)  *Galerina* (13)  *Hypholoma* (13)  *Agrocybe* (12)  *Rhodocollybia* (12)  *Oudemansiella* (12)  *Peniosphora* (11)  *Agaricus* (10)  *Bjerkandera* (10)  *Crepidotus* (9) | *Gymnopilus* (8)  *Dichomitus* (7)  *Laccaria* (7)  *Tricholoma* (7)  *Heterobasidion* (7)  *Ganoderma* (6)  *Hebeloma* (6)  *Omphalotus* (5)  *Leucoagaricus* (4)  *Trametes* (3)  *Rhodonia* (3) | *Cortinarius* (3)  *Fistulina* (2)  *Phlebia* (2)  *Schizophyllum* (1)  *Panaeolus* (1)  *Phanerochaete* (1)  *Fomitopsis* (1)  *Suillus* (1)  *Stereum* (1)  *Pycnoporus cinnabarinus* (3 Aryl Alcohol Quinone Oxidoreductases) |
| **Fungi Type I** (Clade F2) | *Moesziomyces* (1) | *Ustilago* (1) |  |  |
| **Fungi Type III** (Clade F3) | *Podospora* (14)  *Chaetomium* (6)  *Cladorrhinum* (5)  *Thermothelomyces* (4)  *Madurella* (4)  *Lasiosphaeria* (4)  *Schizothecium* (4)  *Cercophora* (3)  *Immersiella* (3)  *Rhypophila* (3)  *Alternaria* (3) | *Corynascus* (3)  *Apodospora* (2)  *Parachaetomium* (2)  *Parathielavia* (2)  *Chaetomidium* (2)  *Lasiosphaeris* (2)  *Staphylotrichum* (2)  *Thozetella* (2)  *Mycothermus* (2)  *Phaeosphaeria* (2) | *Thermothielavioides* (1)  *Cephalotrichum* (1)  *Achaetomium* (1)  *Apiospora* (1)  *Curvularia* (1)  *Decorospora* (1)  *Dichotomopilus* (1)  *Echria* (1)  *Epicoccum* (1)  *Lomentospora* (1) | *Massariosphaeria* (1)  *Ophiobolus* (1)  *Phaeosphaeriaceae* (1)  *Phialemonium* (1)  *Pyrenochaeta* (1)  *Remersonia* (1)  *Scedosporium* (1)  *Thermomyces* (1)  *Triangularia* (1)  *Zopfia* (1) |
| **Bacteria Type II** (Clase B1) | *Geminicoccaceae* (2) |  |  |  |
| **Bacteria Type I** (Clade B2) | *Mycobacterium* (203) |  |  |  |
| **Bacteria Type I** (Clade B3) | *Nonomuraea* (32)  *Actinomadura* (29)  *Streptosporangium* (10)  *Actinobacteria* (7)  *Herbidospora* (6) | *Planomonospora* (4)  *Sphaerisporangium* (4)  *Streptosporangiaceae* (2)  *Planobispora* (2)  *Thermomonospora* (2) | *Acrocarpospora* (1)  *Spongiactinospora* (1)  *Thermocatellispora* (1)  *Agromyces* (1)  *Cellulomonas* (1) | *Rhizocola* (1)  *Yinghuangia* (1)  *Spirillospora* (1)  *Actinocorallia* (1)  *Streptomyces* (1) |
| **Bacteria Type I** (Clade B4) | *Streptomyces* (60) | *Actinobacteria* (11) | *Kitasatospora* (1) |  |
| **Bacteria Type I** (Clade B5) | *Streptomyces* (1) |  |  |  |
| **Bacteria Type II** (Clade B6) | *Saccharothrix* (6)  *Verrucosispora* (4) | *Streptomyces* (3)  *Nocardia* (3) | *Micromonospora* (2)  *Rhodococcus* (2) | *Frankia* (1)  *Actinosynnema* (1) |
| **Bacteria Type III** (Clade B7) | *Streptomyces* (11)  *Bacteroidota* (6)  *Actinomyces* (3)  *Agrobacterium* (3)  *Rhizobium* (3)  *Actinomadura* (2)  *Brocadia* (2) | *Kitasatospora* (2)  *Maribacter* (2)  *Mesorhizobium* (2)  *Actinocrispum* (1)  *Aquimarina* (1)  *Bradyrhizobium* (1)  *Dokdonia* (1) | *Enterobacter* (1)  *Enterococcus* (1)  *Kitasatospora* (1)  *Nitrotoga* (1)  *Pseudomonadota* (1)  *Ruegeria* (1) | *Runella* (1)  *Sphingobacterium* (1)  *Sphingomonadaceae* (1)  *Sphingomonas* (1)  *Subtercola* (1)  *Tenacibaculum* (1) |

**
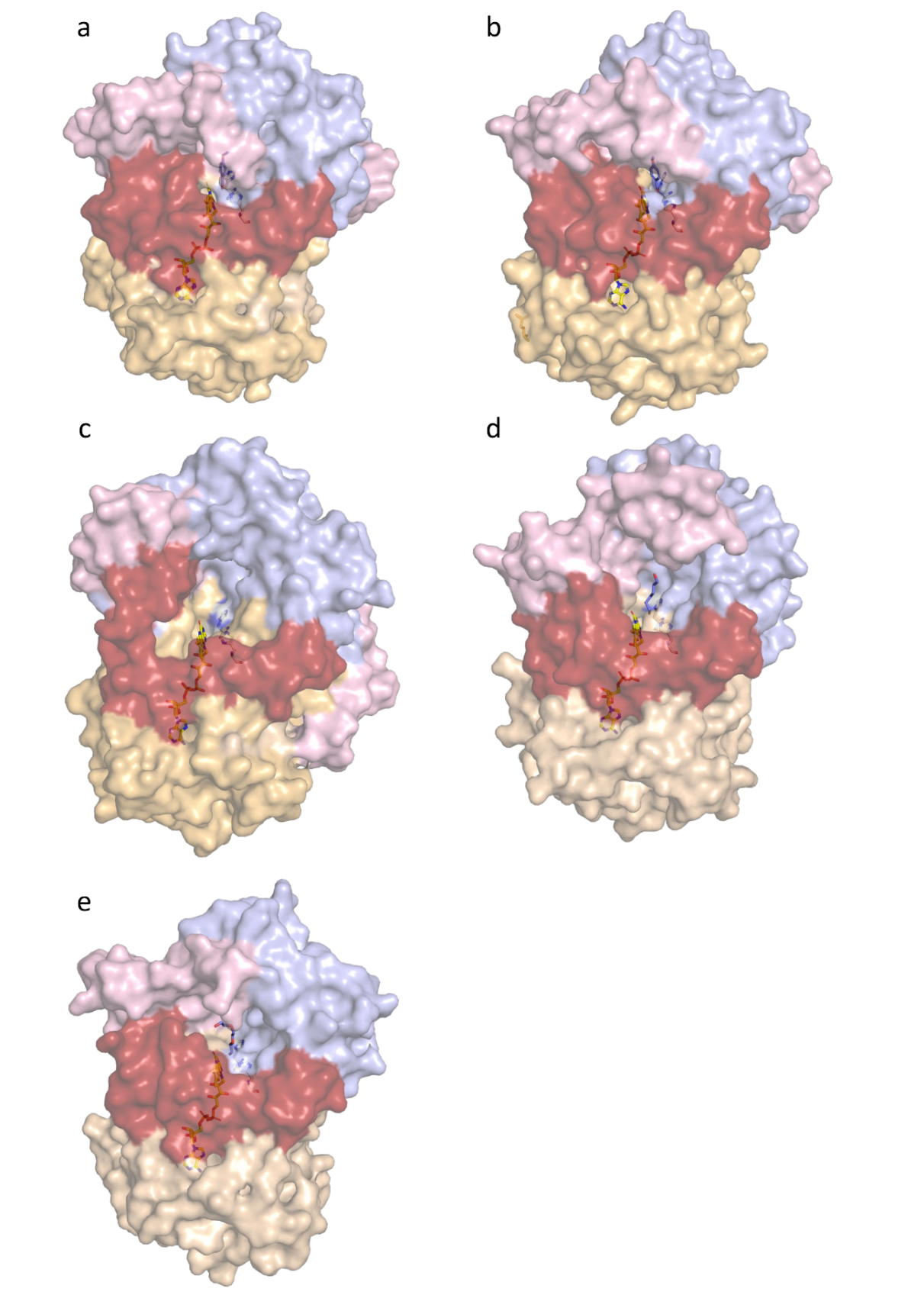
Supplementary Figures**

**Figure S1** Comparative analysis of the active-site accessibility. Panels (a), (b), (c), (d), and (f) illustrate the molecular surfaces of *Pe*AAO (PDB ID: 5OC1), *Ba*AAO (PDB ID: 9AVH), *Mt*AAO (PDB ID: 6O9C), *Sd*AAO (PDB ID: 8RPF), and *Sh*AAO (PDB ID: 82PG), respectively. The substrate-binding domains are highlighted in light blue, the FAD-binding domains in wheat, and the elongated unstructured elements connecting both domains in red. Structural elements distinguishing AAO within the GMC family are depicted in pink (see legend of figure 2 for further details). In all structures, highly conserved histidine residues and FAD cofactors are represented as sticks with carbons atoms in light gray and yellow, respectively. Alcohol molecules (bacterial AAOs) and the 4-methoxybenzoic (*Pe*AAO and *Ba*AAO) are displayed with carbon atoms in blue.
